# Supplementary figures and images for: Distinct Effects of GnRH Immunocastration Versus Surgical Castration on Gut Microbiota
Source: Animals (Basel). 2025 Dec 5;15(24):3512. doi: 10.3390/ani15243512 (PMC12730046; doi:10.3390/ani15243512)

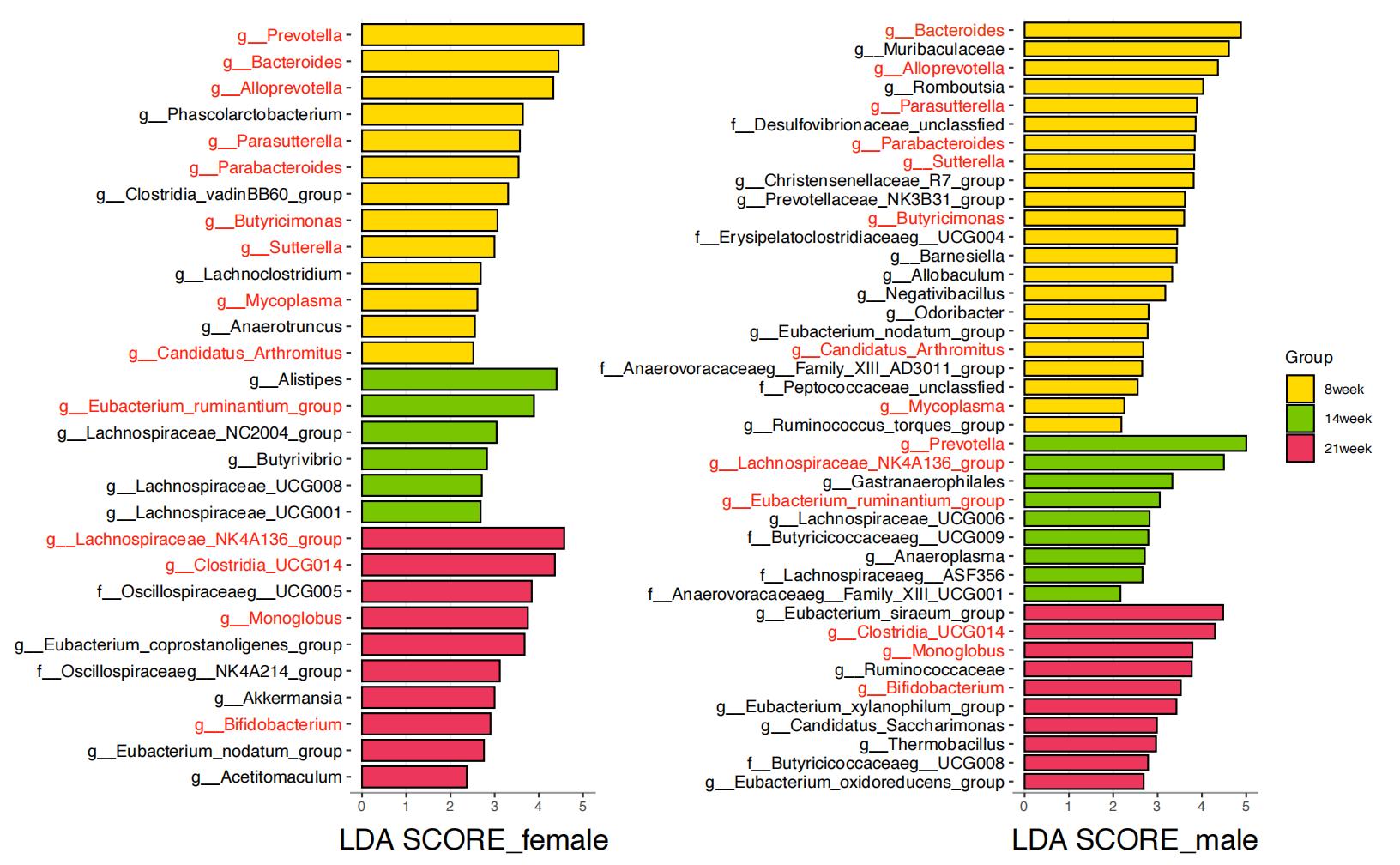

Supplement: Supplementary file 1 [file animals-15-03512-s001.zip › Fig S1 immune castration dynamic lefse lda2.jpg]

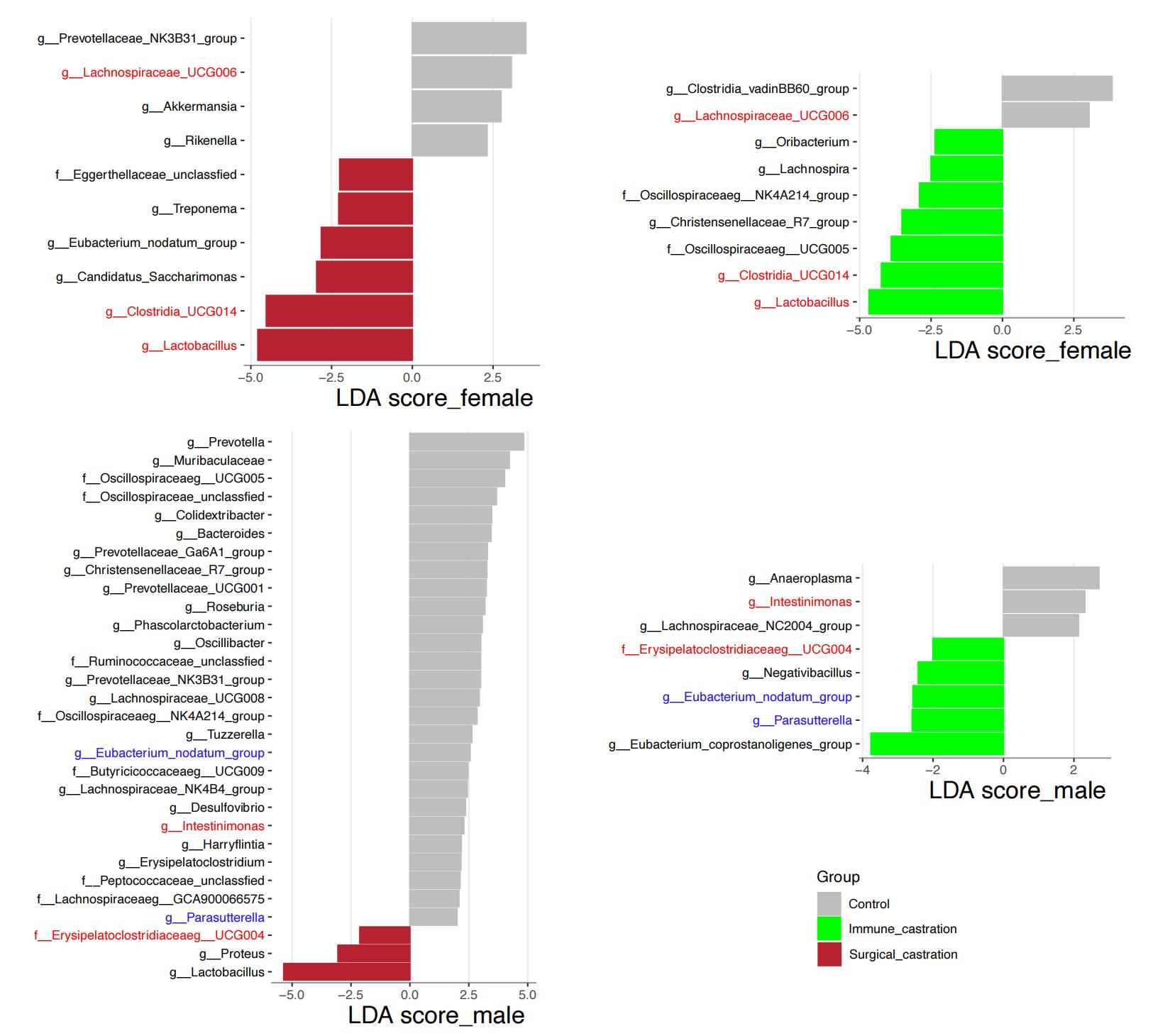

Supplement: Supplementary file 1 [file animals-15-03512-s001.zip › Fig S2 immune and Surgical_castration vs control.jpg]

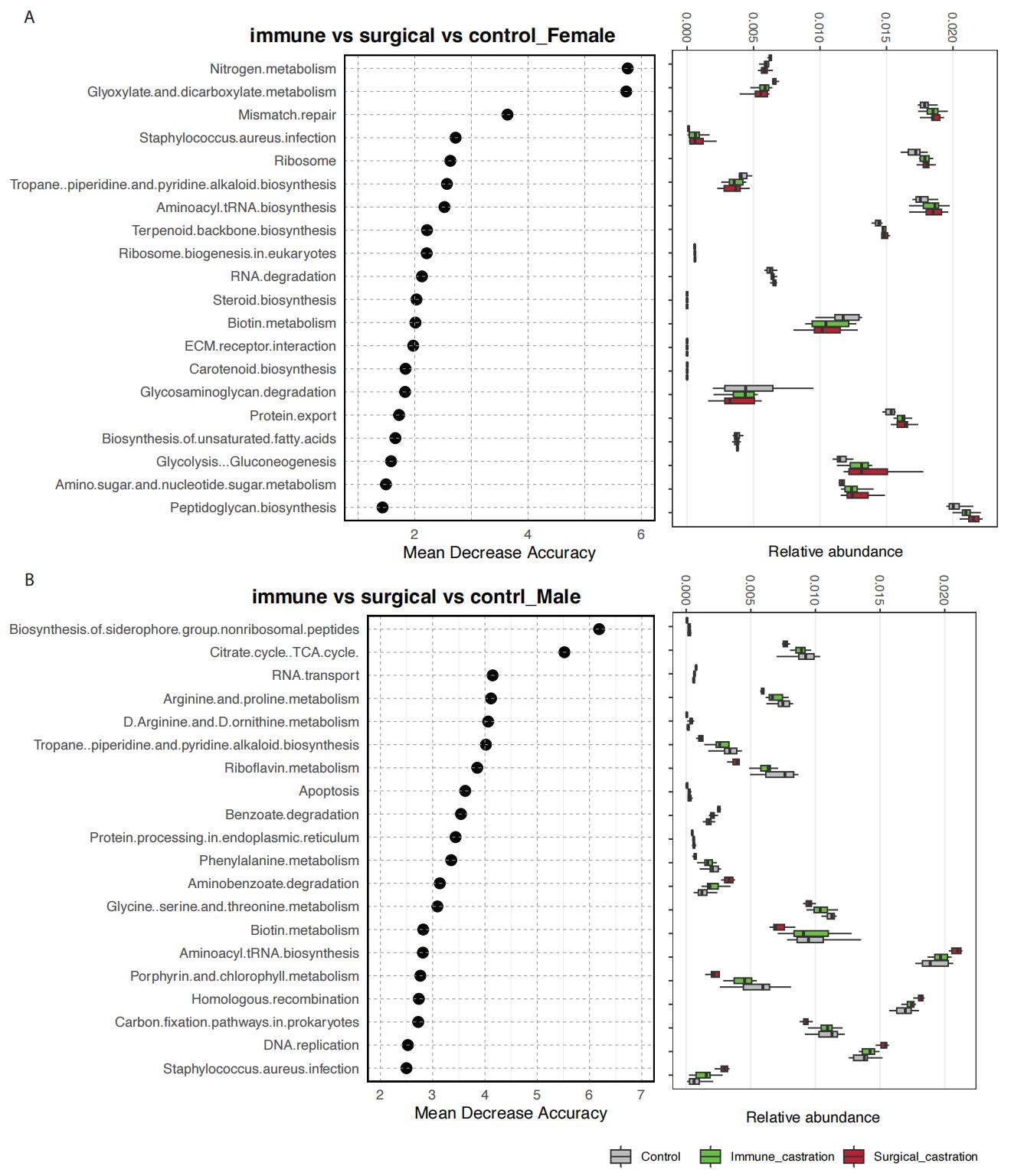

Supplement: Supplementary file 1 [file animals-15-03512-s001.zip › Fig S3 Random forest pathway.jpg]
